# Acoustically Enriched Environment during the Critical Period of Postnatal Development Positively Modulates Gap Detection and Frequency Discrimination Abilities in Adult Rats

**DOI:** 10.1155/2021/6611922

**Published:** 2021-03-12

**Authors:** Kateryna Pysanenko, Natalia Rybalko, Zbyněk Bureš, Daniel Šuta, Jiří Lindovský, Josef Syka

**Affiliations:** ^1^Department of Auditory Neuroscience, Institute of Experimental Medicine, Czech Academy of Sciences, Prague, Czech Republic; ^2^Department of Technical Studies, College of Polytechnics, Jihlava, Czech Republic; ^3^Department of Cognitive Systems and Neurosciences, Czech Institute of Informatics, Robotics and Cybernetics, Czech Technical University, Prague, Czech Republic; ^4^Department of Medical Biophysics and Medical Informatics, Third Faculty of Medicine, Charles University, Prague, Czech Republic

## Abstract

Throughout life, sensory systems adapt to the sensory environment to provide optimal responses to relevant tasks. In the case of a developing system, sensory inputs induce changes that are permanent and detectable up to adulthood. Previously, we have shown that rearing rat pups in a complex acoustic environment (spectrally and temporally modulated sound) from postnatal day 14 (P14) to P28 permanently improves the response characteristics of neurons in the inferior colliculus and auditory cortex, influencing tonotopical arrangement, response thresholds and strength, and frequency selectivity, along with stochasticity and the reproducibility of neuronal spiking patterns. In this study, we used a set of behavioral tests based on a recording of the acoustic startle response (ASR) and its prepulse inhibition (PPI), with the aim to extend the evidence of the persistent beneficial effects of the developmental acoustical enrichment. The enriched animals were generally not more sensitive to startling sounds, and also, their PPI of ASR, induced by noise or pure tone pulses, was comparable to the controls. They did, however, exhibit a more pronounced PPI when the prepulse stimulus was represented either by a change in the frequency of a background tone or by a silent gap in background noise. The differences in the PPI of ASR between the enriched and control animals were significant at lower (55 dB SPL), but not at higher (65-75 dB SPL), intensities of background sound. Thus, rearing pups in the acoustically enriched environment led to an improvement of the frequency resolution and gap detection ability under more difficult testing conditions, i.e., with a worsened stimulus clarity. We confirmed, using behavioral tests, that an acoustically enriched environment during the critical period of development influences the frequency and temporal processing in the auditory system, and these changes persist until adulthood.

## 1. Introduction

Development of the juvenile sensory systems passes through a critical period (CP), a period of increased sensory receptivity and plasticity, during which a particular experience could have a life-long effect. In the case of hearing, proper sound stimulation is essential to establish and maintain normal hearing function. Thus, the auditory experience during the CP plays an important role in the development of perception of behaviorally important sounds and of species-specific communications in different mammalian species including humans.

Rat pups show an onset of hearing at around P11-P12, with the CP extended to the first month of postnatal life [1, 2]. During this period, auditory cortical (AC) neurons demonstrate poor spectral selectivity, weak tonotopic organization, and broad tuning curves, with spectrally broad and temporally extended sideband inhibitory receptive fields in comparison with adult animals, but they appear to be better suited for spectral and temporal integration across a very broad range of acoustic inputs [3]. For these reasons, rat pups are a suitable model for studying the influence of the acoustic environment on the auditory system during its maturation. Absolute acoustical deprivation or inappropriate stimulation during this sensitive developmental period may disrupt proper development of the auditory system and cause permanent alterations of its structure and responsiveness of the auditory brainstem as well as the auditory cortex [1, 3–8]. On the contrary, a well-designed enrichment of the acoustic environment may improve response strength, threshold, selectivity, and latency of the inferior colliculus (IC) [9] and auditory cortex neurons [10, 11], stimulate physiological plasticity in the auditory cortex [12], and improve the decoding performance of vocalizations embedded in noise in the A1 neurons [13]. Moreover, the exposure to an acoustically enriched environment during the critical developmental period can induce permanent changes in the structure of neurons in the central auditory system, such as changes in the dendritic length and volume and density of spines [14].

It has previously been shown that a complex arrangement of enrichment, including acoustic and additional nonacoustic stimulation, can improve the number of correct scores, decrease the reaction time and azimuth deviation in sound-azimuth discrimination test [15], and restore sound frequency discrimination disrupted after early noise exposure in rats to near normal [16]. In these cases, however, the source of the achieved improvements was not entirely an acoustical enrichment.

There are very few studies describing the behavioral consequences of the exposure solely to the acoustically enriched environment during the critical period. Xu et al. [17] demonstrated that in the different sound duration-discrimination tasks, the music-exposed rats acquired the behavior faster than the control rats, supporting the hypothesis that an early auditory enrichment with music enhances the learning ability in an auditory signal-detection task and in a sound duration-discrimination task. On the other hand, rats reared during their postnatal development from day 9 (P9) until day 38 (P38), in either a pulsed-noise stimulus or speech sounds, did not show a significant advantage of consonant, vowel or fricative detection, compared to the control rats [18]. Recently, Homma et al. [13] found that adult rats experiencing moderately loud modulated noise during the auditory critical period showed an improved ability to detect a behavioral signal in noise.

In our previous studies, we described the changes of the responses in the IC [9] and AC neurons [19, 20] after the application of an acoustically enriched environment (AEE) during the critical period of development. We have shown that rats reared in a complex acoustic environment (spectrally and temporally modulated sound reinforced by an active behavioral paradigm with positive feedback) exhibited permanently improved response characteristics; in particular, the neurons of the enriched animals had lower excitatory thresholds, sharper frequency selectivity, and lower proportion of nonmonotonic rate-intensity functions [9, 19]. For a repetitive stimulus, the neurons exhibited a lower spike count variance, indicating a more stable rate coding with a higher degree of similarity across stimulus repetitions. Furthermore, the neurons followed the temporal course of the stimulus more precisely. Importantly, these AEE-induced changes that developed during maturation of the auditory system were permanent and detectable in adulthood. These findings indicate that an acoustically enriched environment during the critical period of postnatal development influences the basic properties of neuronal receptive fields in the IC and AC, which may have implications for the ability to detect and discriminate sounds, and also affects the stochasticity, reproducibility, and fine structure of neuronal spiking patterns [20].

In this study, we applied the same paradigm of AEE developmental exposure to examine whether the plasticity in the auditory system that we previously observed in the neuronal responses of the inferior colliculus and the primary auditory cortex also manifests in behavioral responses to sound stimuli. The experimental paradigm was based on recording and evaluation of the acoustic startle responses (ASR)—reflexive movements in reaction to unexpected auditory stimuli. To accomplish our aim we utilized the ASR and prepulse inhibition (PPI) of ASR, i.e., the inhibition of the ASR induced by the presentation of an acoustical stimulus shortly preceding the startling sound. It was shown previously that PPI of ASR, with proper modifications, provides an efficient and accurate method to assess acoustic discrimination in experimental animals [21]. As the prepulse cues, we used the following: (i) brief noise or pure tone pulses to estimate the animals' sensitivity to sounds of different intensities, (ii) silent gaps embedded in background noise to assess gap detection ability [22–24], or (iii) short-term changes of the background tone frequencies to evaluate the ability of frequency discrimination [21, 25–27]. The ASR reactivity and the PPI of ASR were measured in adult rats reared both in the acoustically enriched environment and under conventional conditions.

## 2. Methods

### 2.1. Experimental Groups

**Figure 1:**
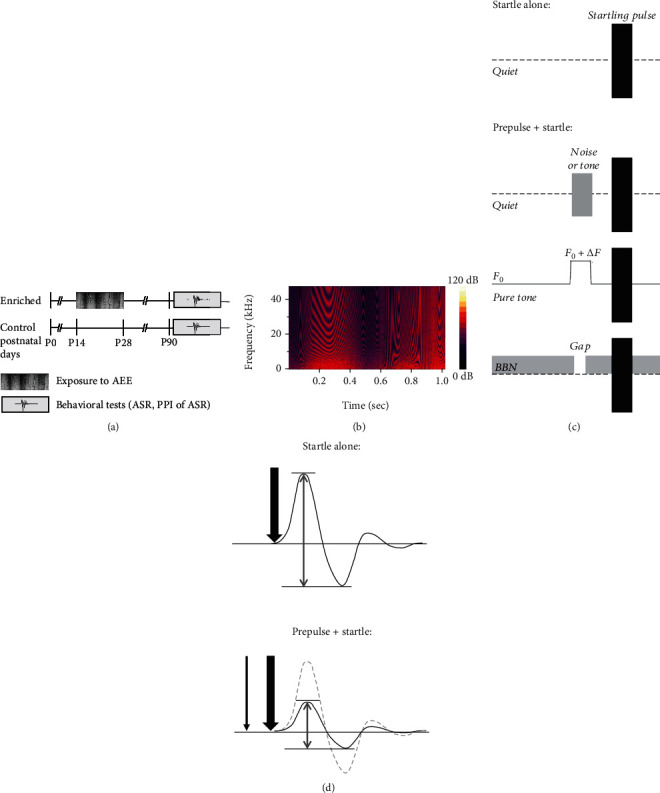
Schematic illustration of the experimental paradigm: (a) time schedule of the experiment; (b) a representative spectrogram of the background rippled noise used for AEE; (c, d) behavioral test arrangement: (c) diagram illustrating trials in testing sessions; (d) representative responses to startle alone trial and startle response in the presence of prepulse.

Female rats of the Long-Evans strain (obtained from the local breeding facility) were used in the study. One group of rats (*enriched*, *n* = 12) was exposed to an acoustically enriched environment (AEE) reinforced with active behavioral feedback for two weeks starting on postnatal day P14 (for more details about the AEE, see [9, 19]). The AEE was presented for 12 hours during the active night period. The stimulus represented a broad-band amplitude-modulated rippled noise (see Figure 1) with temporally variable sinusoidal spectral envelope (frequency range 983–48461 Hz, depth of the spectral ripples 30 dB, amplitude modulated by a low-pass exponential noise envelope with cut-off frequency 2 Hz). The rippled background noise was presented at 55 dB SPL. With the aim to attract the animals' attention to the acoustic stimulation, the noise background was supplemented with several types of embedded target sounds appearing randomly in time (60 dB SPL, 500 ms duration each; spectral contents centered near 6 kHz): pure tone, sawtooth signal, frequency-modulated tone with a modulation depth of 1.5 kHz and modulation frequency of 10 Hz, and a 1/3-octave noise. The frequency-modulated tone triggered the release of a reward—a drop of sweet syrup. The reward output was delayed relative to the triggering sound by 2 s, giving the animals enough time to attend the delivery spout. The drop was always available for approximately 2 s and then fell out of reach of the animals. A group of age-matched rats (*control*, *n* = 12) was raised in standard housing conditions (with an environmental sound level of 35-40 dB SPL, the noise being steady with minimal fluctuations) with no acoustical enrichment.

The rats were housed in a 12 h light/dark schedule and had free access to water and a standard diet. The care and use of animals were approved by the Ethics Committee of the Institute of Experimental Medicine, Academy of Sciences of the Czech Republic, and followed the guidelines of the EU Directive 2010/63/EU for animal experiments.

### 2.2. Behavioral Tests

Behavioral testing of the animals was carried out in adulthood at the age of 3 to 6 months (Figure 1). Behavioral tests were conducted in a sound-attenuated experimental box (Coulbourn Habitest, model E10-21). During the testing, each rat was placed in a wire mesh cage (160 × 85 × 90 mm) on a motion-sensitive platform inside the box. The rat's reflex movements to sound stimuli were detected and transformed to a voltage signal by the load-cell response sensing platform. An amplified voltage signal was acquired and processed using a TDT system 3 with a Real-Time Processor RP 2 (Tucker Davis Technologies, Alachua, Fl) and custom-made software in a Matlab environment. The startle responses were evaluated in 100 ms windows, beginning at the onset of the startling stimulus. The magnitude of the ASR was measured as the maximal peak-to-peak amplitude of transient voltage occurring in the response window. Acoustical stimuli were generated by the TDT system (Real-Time Processor RP 2), amplified and presented via a loudspeaker (SEAS, 29AF/W), and placed inside the chamber above the animal. Stimulus presentation and data acquisition were controlled by a custom-made application in a Matlab environment. Calibration of the apparatus was performed for frequencies between 4 kHz and 32 kHz by a 1/4 inch Brüel & Kjaer 4939 microphone connected to a Brüel & Kjaer ZC 0020 preamplifier and a B&K 2231 sound level meter. During the calibration, the calibrating microphone was positioned at the location of the animal's head in the test cage.

#### 2.2.1. Measurement of Acoustic Startle Reactivity

The startle reactivity of the enriched and control rats was characterized by an amplitude-intensity function, measured to three types of startle stimuli in quiet (broadband noise (BBN) pulse, 6 kHz tone pulse, and 16 kHz tone pulse). Each test session contained six identical blocks, including startling trials consisting of 50 ms duration startle stimuli of different intensities (55, 60, 65, 70, 75, 80, 85, 90, 100, 110, 115, and 120 dB SPL) and a nonstartling trial (for the assessment of baseline activity), which were presented in random order with intertrial intervals varying from 15 to 30 s.

#### 2.2.2. Measurement of Prepulse Inhibition of ASR

For the assessment of PPI of ASR, different prepulse stimuli were used in our study in accordance with the experiment tasks. Each test session of PPI measurement contained eight identical blocks including three trial types: a startle stimulus alone (110 dB SPL BBN burst of 50 ms duration with 0.5 ms rise/fall times), the startle stimulus preceded by a prepulse, and a trial without any stimulation (for the assessment of baseline activity); the trials were presented in random order with the intervals varying from 15 to 30 s.

1. The sensitivity to suprathreshold sounds (BBN, tone of 6 kHz, and tone of 16 kHz) was assessed by measuring the PPI of ASR induced by the given sounds in quiet. The effects of the 50 ms duration sound prepulses of different intensities (10, 20, 30, 40, 50, 60, and 70 dB SPL) were tested in each block of the test session; the interval between startle and prepulse stimulus was 50 ms
2. The assessment of the gap detection ability was performed by measuring the PPI of ASR induced by gaps of various durations embedded in a background BBN. Five gap prepulses of different durations (5, 10, 15, 30, and 50 ms) were used in each test block; the interval between the gap prepulse and the startle pulse (on-on) was 70 ms. The effects of gap prepulses were tested at three levels of background BBN (55 dB SPL, 65 dB SPL, and 75 dB SPL)
3. The assessment of the frequency discrimination ability was performed by measuring the PPI of ASR, induced by a short-term (50 ms) increase of the background tone frequency (*F*_0_) to the value of *F*_0_ + Δ*F* [25]. The interval between the startle and prepulse stimulus was set to 50 ms. The effect of five frequency differences (Δ*F* = 5, 10, 15, 30, or 50% of *F*_0_) of a background tone (*F*_0_ = 6 kHz or 16 kHz) was tested in each block of test sessions. The PPI measurements were performed at three levels of the background tone (55 dB SPL, 65 dB SPL, and 75 dB SPL)

The efficacy of the PPI was expressed as a percentage of the startle amplitude without prepulse: PPI% = (ASR amplitude with prepulse/ASR amplitude without prepulse) × 100%; thus, smaller values of the ASR ratio reflected stronger PPI.

### 2.3. Statistical Analysis

A two-way RM ANOVA and Bonferroni's multiple comparison test were used to determine if the differences among ASR and PPI curves, the same as their individual points, between given experimental groups were significant. All statistical tests were performed in GraphPad Prism (GraphPad Software, La Jolla, CA).

## 3. Results

### 3.1. The Assessment of Startle Reactivity to Noise and Tone

**Figure 2:**
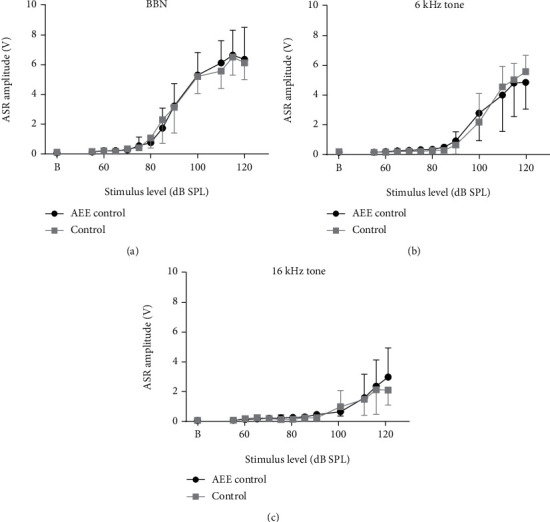
ASR amplitude-intensity functions (mean ± SD) obtained for BBN (a), 6 kHz (b), and 16 kHz (c) startling pulses in enriched (black) and control (gray) animals. (b) Baseline trial without any acoustical stimulation. The curves in each panel are not statistically different (*p* = 0.93, *p* = 0.96, and *p* = 0.53, resp., RM two-way ANOVA).

The comparison of the amplitude-intensity dependence of the ASR for the three tested sounds (BBN, 6 kHz tone, and 16 kHz tone) showed no differences between the enriched and control rats, neither between the whole curves nor at individual intensity points (Figure 2). In both groups, stimulation with BBN pulses had the highest efficacy in startle elicitation. When tone pulses were used as startling stimuli, the magnitude of ASR exhibited a decreasing trend with increasing stimulus frequency in both the enriched and control rats: the 16 kHz tones evoked considerably weaker ASR than the 6 kHz tones, particularly in the range between 100 and 120 dB SPL; the amplitude-intensity curves for these frequencies were significantly different within each experimental group (Figure 2(b) vs.  Figure 2(c), *p* < 0.05 and *p* < 0.001 for enriched and control groups, respectively).

### 3.2. The Assessment of Hearing Functions by PPI Paradigm

#### 3.2.1. Sensitivity to Noise and Tone

**Figure 3:**
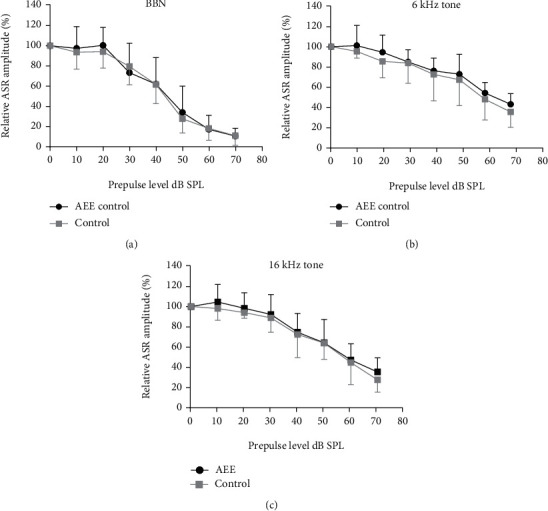
The comparison of PPI of ASR induced by sounds of different intensities in enriched and control rats (mean ± SD): (a) BBN prepulse; (b) 6 kHz prepulse; (c) 16 kHz prepulse; 100% corresponds to the ASR amplitude without prepulse. The curves in each panel are not statistically different (*p* = 0.77, *p* = 0.31, and *p* = 0.34, resp., RM two-way ANOVA).

The estimation of sensitivity to broadband noise and tones of 6 kHz and 16 kHz was performed using a prepulse inhibition of ASR. The comparison of startle PPI induced by BBN or tonal prepulses of various intensities showed no difference in the PPI functions (dependence of the PPI on the prepulse intensity) between the enriched and control animals (Figure 3). These results indicate a similar sensitivity to tested sounds in the whole investigated range of intensities in animals of both groups.

#### 3.2.2. Gap Detection Ability

**Figure 4:**
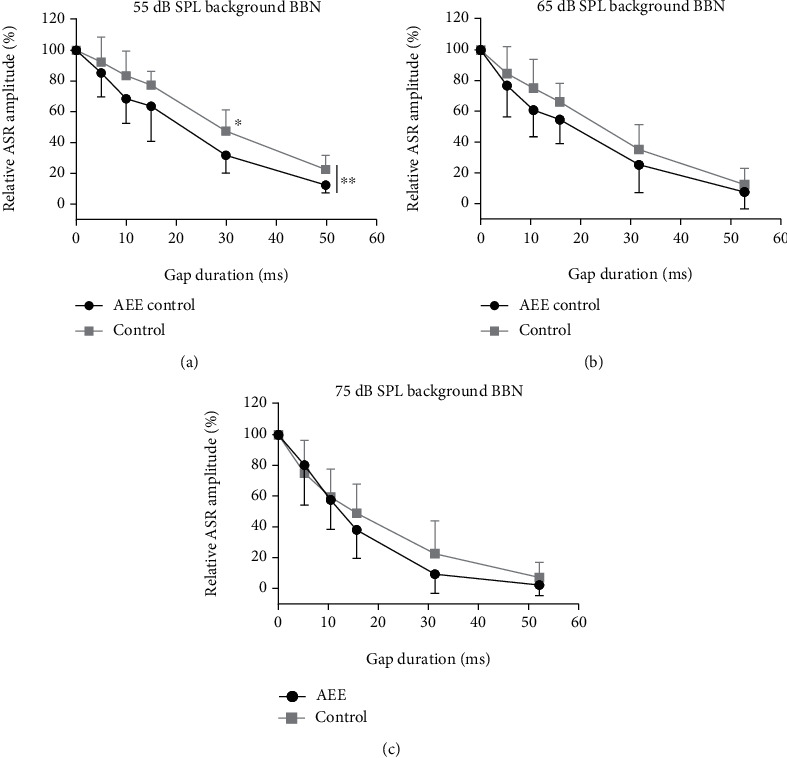
Comparison of the PPI of ASR induced by gaps of different durations in enriched and control animals (mean ± SD) at 55 dB SPL (a), 65 dB SPL (b), and 75 dB SPL (c) of background BBN; 100% corresponds to the amplitude of ASR without gap prepulse; smaller values of ASR amplitude ratio indicate stronger gap-PPI. Statistical significance: (a) ^∗^*p* < 0.05 and ^∗∗^*p* < 0.01; (b) *p* = 0.06; (c) *p* = 0.38, RM two-way ANOVA with the Bonferroni posttest.

To examine the possible effect of the developmental acoustic exposure on the gap detection performance in the adult rats, we studied the PPI of ASR induced by gaps of various durations in a background BBN at three intensity levels (55, 65, and 75 dB SPL). In both experimental groups in all testing conditions, the gap-PPI of ASR increased with the gap prolongation. Significant differences between the enriched and control rats appeared only at the lowest level of background BBN—55 dB SPL (Figure 4), especially for the 30 ms gap duration (47.42% in controls vs. 31.76% in enriched rats, *p* < 0.05, *t* = 2.686, RM two-way ANOVA with the Bonferroni multiple comparison test). With the increase of background BBN intensity from 55 dB to 65 and 75 dB SPL, the differences between the enriched and control groups become marginal.

#### 3.2.3. Frequency Discrimination Ability

**Figure 5:**
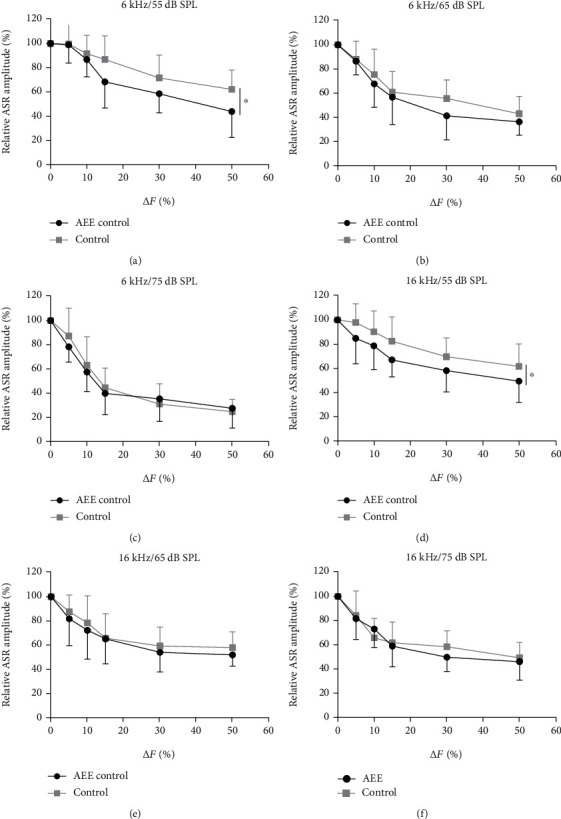
Group-averaged functions showing PPI as a function of Δ*F* of the background pure tone at frequencies of 6 kHz (a–c) and 16 kHz (d–f) (mean ± SD). (a) and (d) illustrate the results at the 55 dB SPL sound intensity, (b) and (e) show data obtained at the 65 dB SPL sound intensity, and (c) and (f) show data obtained at the 75 dB SPL sound intensity. Statistical significance: (a) ^∗^*p* < 0.05; (b) *p* = 0.13; (c) *p* = 0.64; (d) ^∗^*p* < 0.05; (e) *p* = 0.39; (f) *p* = 0.63, RM two-way ANOVA.

To determine whether and how the acoustically enriched rearing affects the frequency discrimination ability, we used the PPI procedure in which the prepulses consisted of a short-term increase of the background tone frequency (increase of *F*_0_ to *F*_0_ + Δ*F* lasting 50 ms). The PPIs of ASR were measured for several frequency differences (Δ*F* = 5, 10, 15, 30, or 50% of *F*_0_), for both the 6 kHz and the 16 kHz tones at three intensity levels (55 dB SPL, 65 dB SPL, and 75 dB SPL).  Figure 5 depicts the PPI of the ASR as a function of the frequency change Δ*F*. The PPIs of ASR grow stronger with the increasing frequency differences in all tested levels of background tone, for both the enriched and control rats. A significantly larger prepulse inhibition of the ASR was found in the enriched rats at the lowest intensity (55 dB SPL) of both the 6 kHz and the 16 kHz background tones. With the increasing background intensity, the detection conditions of frequency changes become easier. Thus, at 65 dB SPL, the improvement of the Δ*F*-detecting ability in the enriched rats was rather small, and at 75 dB SPL, the performance of both groups became comparable for both background tones.

## 4. Discussion

In this study, we examined the effects of an acoustically enriched environment applied during the critical period of the postnatal development on hearing functions of adult rats. A set of behavioral tests based on recording of ASR and its prepulse inhibition was performed. We found differences between the enriched and control animals in the PPI of ASR when prepulse stimuli were silent gaps in the noise or changes in the frequency of the background tone. These differences were significant at lower (55 dB SPL) but not at higher (65-75 dB SPL) intensities of background sounds, i.e., under the more difficult testing conditions.

### 4.1. Approach

To influence the development of the hearing system, we reared rat pups in a synthesized rippled noise for 2 weeks from the hearing onset. The composition of the acoustically enriched environment should be very carefully considered, both from the viewpoint of its content and its intensity. The intensity of the environmental sound must not be too high: our previous experiments showed that rats exposed to loud noise at the onset of hearing showed significant changes of ASR threshold and ASR amplitudes during the following two weeks of life; the effect severity was dependent on the exposure duration [28]. In adulthood, these animals manifested an altered sensation of sound intensity [29] and impaired frequency discrimination and gap detection abilities, even in cases of normal hearing thresholds [25, 30]. Yet even in the case of a moderate level of the environmental sound, the influence may be negative. Several previous studies have demonstrated that exposure to moderate intensity continuous white noise [3], pulsed noise [8, 18, 31, 32], tones [1, 2, 33–35], or frequency-modulated sweeps [36, 37] during development results in degradations of tonotopicity, frequency tuning, or spectral and temporal processing characteristics of cortical and brainstem neurons. A long-term exposure of even adult animals to moderate level noise around 65 dB SPL led to negative cortical changes in temporal and spectral sound processing with significant behavioral impairments of temporal discrimination [38], as well as frequency discrimination in quiet [39]. Consequently, to avoid a pathologic effect of acoustic exposure, we exposed animals to lower levels of sounds (55-60 dB SPL) with thoroughly selected stimuli (wideband, spanning most of the animals' hearing range, modulated both in frequency and in amplitude, and random in many aspects) designed to resemble natural sounds. We did not see any negative changes at any stage of the hearing pathway we studied [9, 19, 20]. Our findings are consistent with studies using as an enriched environment either a very complex enrichment [10, 11, 15, 17] or different spectrotemporally modulated background noises [13] or speech [18] at lower intensities.

### 4.2. Behavioral Testing

In our behavioral tests, we applied three types of stimuli for comparing enriched and control groups of animals: BBN (sufficient strong stimulus, covering wide frequency range (from 0.5 to 25 kHz), 6 kHz pure tone (frequency used during the postnatal exposure to attract the animals' attention to the AEE), and 16 kHz pure tone (selected to be in the range of the lowest hearing thresholds for rats and far enough from the previous stimulus).

The experimental paradigm is based on the use of an acoustic startle response (ASR), which is a reflexive response following the presentation of an unexpected intense stimulus. The circuit mediating the ASR is relatively simple and includes only a few synapses [21, 40, 41]. The structural basis of the ASR includes the cochlear root neurons, neurons of the cochlear nucleus, nucleus of the lateral lemniscus, the caudal pontine reticular nucleus, spinal interneurons, and spinal motor neurons [21, 42–44]. The startle reflex shows several forms of behavioral plasticity including habituation, sensitization, and inhibition that provide important information about central auditory processing. In the current study, we utilize a suppression of ASR amplitude induced through prepulse inhibition with several types of nonstartling stimuli. This allows the perceptual salience of the prepulse stimulus to be determined. Importantly, as the PPI is reflexive, it does not require animal training and therefore it largely eliminates the influence of motivation and attention. The circuit mediating a prepulse on the startle circuit involves the cochlear nucleus, inferior colliculus, superior colliculus, and pedunculopontine tegmental nucleus, which project to the caudal pontine reticular nucleus. The neuronal structures that regulate the PPI-mediating circuit, induced by gaps embedded into the background sounds and changes in the frequency of a background tone, include the limbic cortex, prefrontal cortex, striatum and pallidum, and other central structures including the auditory cortex [21, 24, 44–48]. Therefore, it is particularly the different PPI efficacy in the enriched rats which could reflect experience-dependent plasticity in the central auditory system, caused by an acoustically enriched environment presented during the early postnatal period.

#### 4.2.1. The Effect of AEE on Sensitivity to Sound Stimuli

Exposure of the rat pups to the AEE did not lead to any significant changes in the ASR amplitudes evoked by BBN and both tones, as well as in the PPI of ASR with tone- and BBN-burst prepulses. We therefore did not observe any signs of changes in the processing of signal intensity and loudness perception, which mostly occur as a consequence of an early acoustic trauma [25, 28–30].

#### 4.2.2. The Effect of AEE on Gap Detection Ability

The inhibition of ASR by a short gap in a background BBN (gap-PPI) is widely used for the estimation of the ability to detect gaps [49, 50] to assess auditory temporal resolution [22–24] and is also applied as a screening tool for the presence of tinnitus [51]. In humans, temporal resolution is important for speech recognition, thus the gap detection test represents a clinically feasible measure of speech perception [52, 53], and deficits of gap detection were shown to be well correlated with age-related worsened speech comprehension [54–56].

To reveal a possible effect of a developmental acoustical enrichment on gap detection ability, we used a gap-prepulse paradigm. We evaluated the amounts of ASR inhibition, induced by gaps of different durations in background BBN of different levels (55 dB SPL, 65 dB SPL, and 75 dB SPL), in rats reared either in the AEE or under conventional conditions. Our results showed in all three tested intensity conditions a more pronounced ASR inhibition in the enriched animals. However, a significantly stronger gap-PPI in the enriched rats was observed in the lowest tested intensity of the background BBN (55 dB SPL), i.e., under condition of a more difficult gap detection task. Previously, it has been shown that a decline in the background BBN intensity adds extra complexity to the task and generally leads to a worsened recognition of gaps [57–60]. It is generally accepted that the strength of the startle inhibition reflects the perceptual salience of the prepulse [24, 61–63]. We can therefore conclude that the detection ability of the same gap is higher in the enriched rats in comparison with the controls, particularly in situations of reduced audibility of acoustic stimulation. Similarly with previous studies [24, 30, 63], we experienced increases of inhibition efficacy with prolonging gap durations in both animal groups. A weak inhibition of ASR induced by 5 ms gap in both groups indicated that this stimulus was hardly noticed by all the rats. The gaps of longer duration (10-30 ms) produced a stronger and reliable inhibition of ASR in all the rats; however, the improvement in gap-PPI was more pronounced in the enriched rats. The performance for the 30 ms gaps in 55 dB SPL background BBN was significantly better in the enriched rats. A further increase of the gap duration of up to 50 ms made the task much easier and reduced the difference in the gap-PPI performance between the enriched and control groups, indicating similar perceptual salience of this gap duration. An increase of background BBN intensity to 65 dB SPL and 75 dB SPL led to improvement of gap-PPI performance in both groups and to a diminished difference in gap-PPI between the enriched and control animals. We may thus conclude that the rearing of the animals in the AEE improved their gap detection ability. The improvement is most pronounced particularly under the more difficult conditions of the gap detection task (gaps of middle duration embedded in low-intensity BBN). These findings can serve as evidence of the positive changes in auditory temporal processing, due to developmental acoustical enrichment, as opposed to impairments of the gap detection ability observed in adult rats which were exposed to loud noise at the onset of hearing [7, 30].

#### 4.2.3. The Effect of AEE on Frequency Discrimination

In our previous studies, we showed that the AEE paradigm we applied led to an improvement of frequency selectivity of the IC and AC neurons [9, 19]. We thus hypothesized that the AEE could improve the frequency discrimination ability of exposed animals measured by behavioral technique. We used the modified method of the PPI of ASR to assess auditory frequency discrimination through the ability to detect short-term changes in the frequency of a background tone which served as the prepulse stimulus [21, 25–27]. The enriched rats manifested an overall improvement in the detection of frequency changes. A significantly stronger prepulse inhibition of the ASR, which reflects a more effective detection of the frequency changes, was found in the enriched rats at the lowest tested intensity (55 dB SPL) of both the 6 kHz and 16 kHz background tones. The significant differences between the enriched and control rats were not observed when measurements were performed at higher intensities (65 or 75 dB SPL) of background tones. The increasing of the background sound intensity improves the perceptual clarity of the frequency changes, the test became easier, and the superiority of the enriched animals decreased, similarly to the results obtained in the gap detection tests. Importantly, the improvement of frequency discrimination ability was present at both tested frequencies, so the effect of the exposure is not specific to the frequency of the supplemental sounds used in the enrichment as an attention-getter but is more generalized. Analogously, Homma et al. [13] showed that after rearing rats in moderately loud spectrotemporally modulated background noises, behavioral benefits of noise exposure were not narrowly focused on the statistics of the exposure noise.

Frequency discrimination is one of the fundamental features of the auditory system. The ability to detect and encode fast changes in the spectral characteristics of sound is essential for vocal communication [26]. Human studies showed that patients with congenital or early developed mild or moderate sensorineural hearing loss often show impairments in their ability to perceive spectral differences between sounds [64, 65], which may contribute to marked deficits in the development of language, speech perception, and literacy. On the other hand, Au et al. [66] described that a regular exposure to a foreign language during early childhood, even without its active usage, results in a more native-like accent when the subjects learn the language as adults.

It has been long known that from the third trimester of gestation, human fetuses not only respond to pure tones [67] or noise [68] stimulation but also are able to discriminate between different pure tones or different speech sounds [69]. This finding confirms that the early auditory environment, even during late gestation, but especially for infants born premature, is crucial. Therefore, early experience of complex and well-structured acoustic environment has a substantial impact on the auditory perception and perceptual behavior and could prevent development of the problems with speech perception and language acquisition, in particular in acquiring the phonological and grammatical patterns of the language [70–72].

## 5. Conclusions

The results of our study indicate that a properly arranged, acoustically rich, and stimulating environment applied during the critical period of auditory system development has the power to improve frequency discrimination ability and temporal resolution. As such, they represent behavioral evidence of developmental experience-dependent plasticity in the auditory system, which has a long-lasting effect and is preserved to adulthood. The results may have implications also for the human neonatal care, as frequency discrimination and temporal processing of an acoustic stimulus in the auditory system are essential for speech perception and understanding, especially in the challenging listening conditions.

## Data Availability

All data used during the study are available from the corresponding author by request.
